# Prevalence, risk factors, and outcomes associated with delayed second doses of antibiotics in sepsis at a large academic medical center

**DOI:** 10.1017/ash.2023.480

**Published:** 2023-11-10

**Authors:** Meghan E. Cook, Brian R. Schuler, Michael J. Schontz, Kevin C. McLaughlin, Kenneth E. Lupi, Jeremy R. DeGrado, Chanu Rhee

**Affiliations:** 1 Department of Pharmacy, Brigham and Women’s Hospital, Boston, MA, USA; 2 Department of Population Medicine, Harvard Medical School/Harvard Pilgrim Health Care Institute, Boston, MA, USA; 3 Division of Infectious Diseases, Department of Medicine, Brigham and Women’s Hospital, Boston, MA, USA

## Abstract

**Objective:**

To evaluate the prevalence, risk factors, and clinical impact of delays in second doses of antibiotics in patients with sepsis.

**Design:**

Single-center, retrospective, observational study.

**Setting:**

Large teaching hospital.

**Patients:**

Adult patients who triggered an electronic sepsis alert in the emergency department (ED), received ≥2 doses of vancomycin or an antipseudomonal beta-lactam, and were discharged with an ICD-10 sepsis code.

**Methods:**

We assessed the prevalence of delays in second doses of antibiotics by ≥25% of the recommended dose interval and conducted multivariate regression analyses to assess for risk factors for delays and in-hospital mortality.

**Results:**

The cohort included 449 patients, of whom 123 (27.4%) had delays in second doses. In-hospital death occurred in 31 patients (25.2%) in the delayed group and 71 (21.8%) in the non-delayed group (*p* = 0.44). On multivariate analysis, only location in a non-ED unit at the time second doses were due was associated with delays (OR 2.75, 95% CI 1.20–6.32). In the mortality model, significant risk factors included malignant tumor, respiratory infection, and elevated Sequential Organ Failure Assessment (SOFA) score but not delayed second antibiotic doses (OR 1.19, 95% CI 0.69–2.05). In a subgroup analysis, delayed second doses were associated with higher mortality in patients admitted to non-intensive care units (ICUs) (OR 4.10, 95% CI 1.32–12.79).

**Conclusions:**

Over a quarter of patients with sepsis experienced delays in second doses of antibiotics. Delays in second antibiotic doses were not associated with higher mortality overall, but an association was observed among patients admitted to non-ICUs.

## Introduction

Despite advances in recognition and treatment, sepsis remains a leading cause of mortality worldwide.^1,2^ Best practice guidelines and national quality measures emphasize the importance of timely administration of initial antibiotics in patients with sepsis.^1–3^ These recommendations are supported by numerous observational studies demonstrating associations between delays in antibiotics and higher mortality, particularly in patients with septic shock.^4–9^ However, ensuring appropriate timing of subsequent antibiotics is left largely unaddressed.^1–3^

Previous retrospective studies have suggested that delays in second doses of antibiotics are common in patients with sepsis and may be associated with worse outcomes.^10–12^ Additional data are needed, however, given that there are differences across hospitals and regions in sepsis treatment patterns and there have been conflicting findings on the association between these delays and mortality.^10–15^ We therefore sought to assess the prevalence, risk factors, and outcomes associated with delayed second doses of antibiotics in patients with sepsis at a large academic medical center.

## Methods

### Study design and patient cohort

We conducted a retrospective study of adult patients (≥18 years old) admitted with sepsis who were started on vancomycin and/or an antipseudomonal beta-lactam in the emergency department (ED) of Brigham and Women’s Hospital in Boston, Massachusetts, between January 1, 2018 and December 31, 2019. Patients were included in the analysis if they 1) had an ICD-10 discharge diagnosis code for sepsis, severe sepsis, or septic shock, 2) were flagged for suspected sepsis by an institutional electronic health record (EHR)-based best practice alert (BPA) (triggered by signs of possible infection and abnormal physiologic signs; Supplementary Table 1) in the ED, and 3) received ≥2 doses of vancomycin or an antipseudomonal beta-lactam with the first dose within 6 hours of the BPA. We focused on these antibiotics as antipseudomonal beta-lactams often form the backbone of broad-spectrum coverage in septic patients and additional methicillin-resistant *Staphylococcus aureus* (MRSA) coverage with vancomycin is often used empirically for severely ill patients; furthermore, both classes of antibiotics act by a time-dependent killing mechanism. Exclusion criteria included transfer from another acute care hospital or death within 24 hours of ED presentation (to avoid including severely ill patients in whom the timing of subsequent antibiotic administration was unlikely to have affected their clinical outcome). The study was approved by Mass General Brigham Institutional Review Board on September 8, 2021 (protocol 2021P002488). Informed consent was waived, and study procedures were compliant with the ethical standards of Mass General Brigham and with the Helsinki Declaration of 1975, as recently amended.

Data collected included patient demographics, pertinent medical history, baseline laboratory values, admitting unit, location at the time of second dose administration, time in the ED, antibiotics administered, Sequential Organ Failure Assessment (SOFA) score (using the worst physiologic variables within 24 hours of the inclusion BPA), and presence of ≥2 systemic inflammatory response syndrome (SIRS) criteria at the time of the inclusion BPA firing. Type of infection was identified using a combination of microbiology data on admission and initial provider notes. Compliance with a modified 3-hour bundle was assessed (lactate measurement, blood cultures, and administration of an antipseudomonal beta-lactam) within 3 hours of the inclusion BPA. The initiation of stress dose steroids, methylene blue, mechanical ventilation, or continuous renal replacement therapy (CRRT) within 24 hours of the inclusion BPA was collected. In addition, the initiation of vasopressors within 12 hours of the inclusion BPA and the number of vasopressors required within 24 hours of the inclusion BPA were recorded as well as durations of vasopressor use, mechanical ventilation, CRRT and lengths of ED, intensive care unit (ICU), and hospital stay.

### Outcomes and statistical analysis

We assessed the prevalence of delays in second doses of vancomycin or antipseudomonal beta-lactams, defined as administration at an interval ≥25% of the recommended dosing interval according to prior studies (e.g., a second dose administered at ≥10 hours rather than the recommended dosing interval of 8 hours would be considered delayed).^10,11^ Recommended dosing intervals were determined using institution-specific dosing guidelines based on creatinine clearance using initial serum creatinine values (Supplementary Tables 2 and 3). Vancomycin delays in patients with renal insufficiency were determined using the methodology outlined in Supplementary Figure 1. Two pharmacists assessed dosing intervals for each patient to ensure concordance in adjudication of antibiotic delays. Any disagreements on the appropriate recommended dosing interval were discussed among the two adjudicators until a consensus was made. At our institution, antipseudomonal beta-lactam administration time defaults to extended infusion (typically 2–4 hours) in the EHR and smart pump drug library. Hospital policy specifies that first doses can be administered over 30 minutes in certain situations, such as when patients have limited intravenous access.

We assessed in-hospital mortality rates in the delayed versus non-delayed second antibiotic dose groups, as well as several exploratory outcomes including ICU mortality, ICU and hospital lengths of stay, initiation and duration of vasopressors, mechanical ventilation, CRRT, and rates of discharge to hospice. We further assessed for risk factors associated with second antibiotic dose delays and the association between delays and in-hospital mortality rates using multivariate logistic regression models. Based on prior literature, we identified a priori the following variables to include in the model assessing risk factors for delays: time in the ED (in hours), location at the time of second dose administration, SOFA score within 24 hours of the inclusion BPA, and antipseudomonal beta-lactam recommended dosing interval of 6–8 hours versus >8 hours.^10–12^ Additional variables were included if individual *p*-values were <0.2 on univariable analysis and included bloodstream infection, admission to an ICU, and initial serum creatinine. For the mortality analysis, the following variables were included a priori based on prior literature: delayed second doses of antibiotics and SOFA score within 24 hours of the inclusion BPA.^10–12^ The following variables were also included in the mortality model if *p*-values were ≤0.05 on univariable analysis: body mass index (BMI), malignant tumor, respiratory source of infection, admission to a non-ICU unit, location at the time of second dose administration, time in the ED, initial lactate level, and initiation of stress dose steroids within 24 hours of the inclusion BPA. Based on prior work suggesting that the association between second antibiotic dose delays and mortality may be mediated by severity-of-illness, we also stratified the mortality analysis in patients admitted from the ED to ICU versus non-ICUs.^12^ All outcomes and covariates were abstracted by medical record review and manually entered into REDCap.^16^

Nominal data were analyzed using the χ^2^ test, and continuous data were analyzed using Mann–Whitney U (nonparametric data) and Studentʼs *t* (parametric data) tests. Data were expressed as incidence or median [interquartile range], as appropriate. We considered *p* < 0.05 to be statistically significant and used two-tailed tests. All analyses were conducted in Stata (version 17.0; StataCorp).

## Results

### Study cohort characteristics and crude outcomes

**Figure 1.**
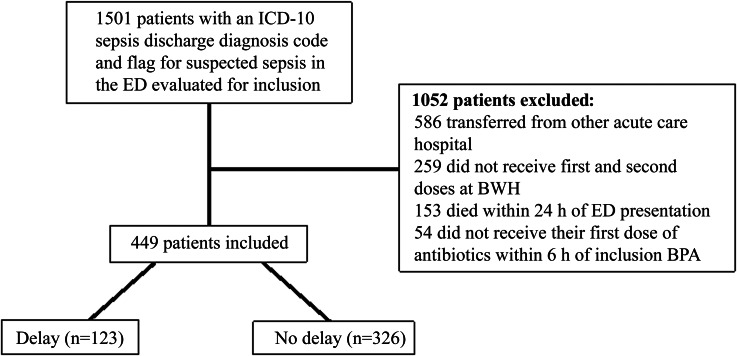
Study flowchart. Abbreviations: ED, emergency department; BWH, Brigham and Women’s Hospital; BPA, best practice alert.

A total of 1,501 patients were evaluated for inclusion in the analysis of which 449 patients met inclusion criteria. Most patients were excluded due to being transferred from a different acute care hospital. Of the 449 patients included, 123 (27.4%) had a delay in second doses of antibiotics (Figure 1). Patient characteristics were similar in both groups except that those in the non-delayed second dose group had significantly higher initial median serum creatinine (1.4 mg/dL vs 1.2, *p* = 0.02) and a trend toward higher initial median lactate levels (2.7 mmol/L vs 2.4, *p* = 0.05) (Table 1). There were no significant differences in compliance with the modified 3-hour bundle along with type and volume of fluid administered between the delayed and non-delayed groups (Table 1 and Supplementary Table 4).

**Table 1.** Baseline characteristics of the study groups

Variable	Delayed group (*n* = 123)	Non-delayed group (*n* = 326)	*p*
Gender, male^a^	64 (52.0)	165 (50.6)	0.79
Age, years^b^	66.0 [52.5–77.5]	68.0 [57.3–76.0]	0.24
Weight, kg^b^	74.4 [58.7–85.8]	72.6 [60.9–84.6]	0.97
BMI, kg/m2^b^	25.1 [21.3–29.9]	25.8 [21.8–30.2]	0.44
Race/ethnicity^a^			0.57
White	77 (62.6)	209 (64.1)	0.77
Asian	12 (9.8)	19 (5.8)	0.14
African American/Black	19 (15.4)	55 (16.9)	0.72
Hispanic	9 (7.3)	31 (9.5)	0.47
Not provided	6 (4.9)	12 (3.7)	0.56
Comorbidities^a^			
Heart failure	25 (20.3)	62 (19.0)	0.75
ESRD	11 (8.9)	19 (5.8)	0.24
Malignant tumor	60 (48.8)	151 (46.3)	0.64
Type of infection^a^			
Respiratory	43 (35.0)	96 (29.4)	0.26
Urinary	24 (19.5)	68 (20.9)	0.75
Intra-abdominal	28 (22.8)	53 (16.3)	0.11
SSTI	11 (8.9)	28 (8.6)	0.91
Bone/joint	2 (1.6)	9 (2.3)	0.49
CNS	0 (0.0)	0 (0.0)	
Bacteremia	38 (30.9)	130 (39.9)	0.08
Endocarditis	1 (0.8)	2 (0.6)	0.82
Unknown	13 (10.6)	43 (13.2)	0.45
Admitting unit^a^			
Non-ICU	49 (39.8)	122 (37.4)	0.64
MICU	54 (43.9)	156 (47.9)	0.45
SICU	6 (4.9)	20 (6.1)	0.61
Thoracic ICU	3 (2.4)	3 (0.9)	0.21
CCU	7 (5.7)	22 (6.7)	0.68
BT ICU	1 (0.8)	1 (0.3)	0.47
Neuro ICU	3 (2.4)	2 (0.6)	0.10
Location at the time of second dose administration^a^			<0.01
ED	12 (9.8)	56 (17.2)	0.05
ICU	49 (39.8)	165 (50.6)	0.04
Non-ICU	62 (50.4)	105 (32.2)	<0.01
Time in ED, hours^b^	6.7 [5.3–9.2]	6.3 [4.7–8.8]	0.11
Time to inclusion BPA from ED arrival, hours^b^	1.5 [0.8–2.3]	1.4 [0.5–2.4]	0.67
Blood cultures^a^			
Positive	43 (35.0)	140 (43.0)	0.12
Negative	79 (64.2)	184 (56.4)	0.14
Not drawn	1 (0.8)	2 (0.6)	0.82
Restricted antimicrobial ordered^a^	15 (12.2)	40 (12.3)	0.98
First dose	11 (8.9)	20 (6.1)	0.30
Second dose	4 (3.3)	20 (6.1)	0.23
Antimicrobial administered^a^			
Antipseudomonal beta-lactam	114 (92.7)	301 (92.3)	0.90
Vancomycin	91 (74.0)	225 (69.0)	0.30
Both	82 (66.7)	200 (61.3)	0.30
Antipseudomonal beta-lactam administered before vancomycin^a,c^			
First dose	65 (79.3)	157 (78.5)	0.89
Second dose	68 (82.9)	182 (91.0)	0.05
SOFA score^b^	6.0 [4.0–10.0]	7.0 [4.0–10.0]	0.45
Compliance with 3-h modified bundle^a^	34 (27.6)	102 (31.3)	0.45
Met SIRS criteria^a^	79 (64.2)	225 (69.0)	0.33
Initial serum creatinine, mg/dL^b^	1.2 [0.7–2.2]	1.4 [1.0–2.3]	0.02
Initial calculated creatinine clearance, mL/min^b^	54.0 [27.5–95.0]	42.0 [27.0–65.0]	0.01
Initial lactate, mmol/L^b^	2.4 [1.8–3.4]	2.7 [1.9–4.3]	0.05
Repeat lactate, mmol/L^b,d^	1.7 [1.2–3.1]	2.1 [1.4–3.4]	0.09
Initiation of stress dose steroids within 24 h of inclusion BPA^a^	32 (26.0)	82 (25.2)	0.85

Note. BMI, body mass index; ESRD, end-stage renal disease; SSTI, skin and soft tissue infection; CNS, central nervous system; ICU, intensive care unit; MICU, medical intensive care unit; SICU, surgical intensive care unit; CCU, coronary care unit; BT ICU, burn trauma intensive care unit; BPA, best practice alert; ED, emergency department; SOFA, Sequential Organ Failure Assessment SIRS, systemic inflammatory response syndrome.

a Data are presented as *n* (%).

b Data are presented as median [interquartile range].

c Patients who received both an antipseudomonal beta-lactam and vancomycin.

d Not available for 4 subjects in the delayed group and 12 subjects in the non-delayed group.

In the delayed group, 77 (62.6%) of patients had a delay in an antipseudomonal beta-lactam, 57 (46.3%) in vancomycin and 11 (8.9%) in both. For patients with delays, the median delay was 3.4 hours [IQR 2.5–4.8] for antipseudomonal beta-lactams and 13.4 hours [IQR 5.2–25.7] for vancomycin.

On crude analysis, there was no significant difference in in-hospital mortality between the delayed and non-delayed groups (25.2% vs 21.8%, *p* = 0.44) (Table 2). There was also no difference in ICU mortality (23.0% vs 25.5%, *p* = 0.67) or ICU length of stay (3 days vs 3, *p* = 0.60). Hospital length of stay and the durations of vasopressor use, mechanical ventilation, and CRRT were not significantly different between groups. More patients in the non-delayed group required two vasopressors within 24 hours of the inclusion BPA (14.1% vs 6.5%, *p* = 0.03).

**Table 2.** Mortality and other outcomes

Outcome	Delayed group (*n* = 123)	Non-delayed group (*n* = 326)	*p*
In-hospital mortality^a^	31 (25.2)	71 (21.8)	0.44
Discharge to hospice^a^	7 (5.7)	18 (5.6)	0.94
ICU mortality^a^	17 (23.0)	52 (25.5)	0.67
ICU length of stay (days)^b^	3.0 [1.0–6.0]	3.0 [2.0–6.0]	0.60
Hospital length of stay (days)^b^	7.0 [4.0–13.0]	8.0 [5.0–14.0]	0.30
Vasopressors initiated within 12 h of inclusion BPA^a^	53 (43.1)	161 (49.4)	0.23
Number of vasopressors used within 24 h of inclusion BPA^a^			0.26
1	32 (26.0)	82 (25.2)	0.85
2	8 (6.5)	46 (14.1)	0.03
3	8 (6.5)	23 (7.1)	0.84
≥4	5 (4.1)	10 (3.1)	0.60
Duration of vasopressor use (days)^b^	2.0 [1.0–3.0]	2.0 [1.0–3.0]	0.40
Mechanical ventilation initiated within 24 h of inclusion BPA^a^	18 (14.6)	64 (19.6)	0.22
Duration of mechanical ventilation (days)^b^	3.5 [2.0–7.5]	3.0 [1.0–7.0]	0.56
CRRT initiated within 24 h of inclusion BPA^a^	3 (2.4)	4 (1.2)	0.36
Duration of CRRT (days)^b^	2.0 [1.5–4.0]	3.5 [2.8–5.0]	0.28

Note. ICU, intensive care unit; BPA, best practice alert; CRRT, continuous renal replacement therapy.

a Data are presented as *n* (%).

b Data are presented as median [interquartile range].

### Multivariate models for risk factors and outcomes of delayed second antibiotic doses

On multivariate analysis, only location in a non-ED unit at the time second doses were due was significantly associated with delays in second antibiotic doses (OR 2.75, 95% CI 1.20–6.32) (Table 3). In the multivariate regression model assessing mortality, there was no association between delayed second doses and in-hospital death (OR 1.19, 95% CI 0.69–2.05). Significant predictors of mortality included malignant tumor (OR 2.11, 95% CI 1.26–3.53), respiratory infection (OR 1.91, 95% CI 1.15–3.17), and elevated SOFA score (OR 1.16 per 1-point SOFA score increase, 95% CI 1.08–1.25); increased body weight was associated with a reduction in mortality (OR 0.99 per 1-point BMI increase, 95% CI 0.98–1.00) (Table 4).

**Table 3.** Regression analysis evaluating for risk factors for delays in second doses

Variable	OR (95% CI)	*p*
Bacteremia	0.68 (0.43–1.07)	0.10
Admission to ICU	0.91 (0.53–1.56)	0.73
Time in ED (per hour)	1.03 (0.99–1.08)	0.16
Non-ED location at time second dose due	2.75 (1.20–6.32)	0.02
Initial serum creatinine	0.91 (0.78–1.06)	0.23
Antipseudomonal beta-lactam interval of 6 to 8 h (vs >8 h)	1.00 (1.00–1.00)	0.77
SOFA score (per 1 point increase)	1.00 (0.94–1.07)	0.91

Note. ICU, intensive care unit; ED, emergency department; SOFA, Sequential Organ Failure Assessment.

**Table 4.** Regression analysis evaluating the association between delayed second doses of antibiotics and in-hospital mortality

Variable	OR (95% CI)	*p*
Delay in second dose	1.19 (0.69–2.05)	0.52
Weight (per 1 kg/m^2^ unit increase)	0.99 (0.98–1.00)	0.04
Malignant tumor	2.11 (1.26–3.53)	<0.01
Respiratory infection	1.91 (1.15–3.17)	0.01
Admission to non-ICU	0.55 (0.27–1.13)	0.10
Time in ED (per hour)	0.96 (0.89–1.04)	0.37
SOFA score (per 1 point increase)	1.16 (1.08–1.25)	<0.01
Non-ED location at time second dose due	1.55 (0.57–4.24)	0.39
Initiation of stress dose steroids within 24 h of inclusion BPA	1.24 (0.72–2.13)	0.45

Note. BMI, body mass index; ICU, intensive care unit; ED, emergency department; SOFA, Sequential Organ Failure Assessment; BPA, best practice alert.

On subgroup analysis for patients admitted to ICU (*n* = 278) versus non-ICUs (*n* = 171), in-hospital mortality occurred in 22 (29.7%) of the delayed group and 64 (31.4%) of the non-delayed group in ICU patients (*p* = 0.79), and 9 (18.4%) of the delayed group and 7 (5.7%) of the non-delayed group (*p* = 0.01) in non-ICU patients. Multivariate regression confirmed an association between second dose delays and mortality in non-ICU patients (OR 4.10, 95% CI 1.32–12.79) but not in patients admitted to the ICU (Supplementary Tables 5 and 6).

## Discussion

In this retrospective study of patients with sepsis treated with vancomycin and/or antipseudomonal beta-lactams in the ED at a large academic medical center, approximately one in four patients experienced a delay in second doses of antibiotics by at least 25% of the recommended dosing interval. Delays were more likely in patients who had transferred out of the ED prior to second doses being due, suggesting transitions of care as a contributing factor. We did not find significant differences in outcomes between the delayed and non-delayed groups in our primary analysis but did observe higher mortality associated with delayed second doses in patients admitted from the ED to non-ICUs.

The prevalence of delays observed in this analysis is similar to the rates observed in the analyses conducted by Leisman et al, Lykins et al, and Kemmler et al (27% vs 33%, 31%, and 21%, respectively).^10,11,17^ These studies used the same definition of delay with a threshold of ≥25% of the recommended dosing interval. In contrast, Parks-Taylor et al used a threshold of delay by >1 hour resulting in more than half of their patient population experiencing a delay in the second dose of antibiotics.^12^ Regardless, these studies performed in different populations and health systems collectively provide convincing evidence that delays in second antibiotic doses are very common in patients with sepsis.

We observed a greater likelihood of delays in patients receiving their second antibiotic doses if they had already departed the ED by the time the second dose was due. This may reflect issues related to hand-offs and/or new orders from receiving teams; transitions from the ED to inpatient settings have previously been associated with adverse events in non-sepsis populations.^18^ This marks a potential quality improvement opportunity that could be addressed via educational initiatives and/or clinical decision support tools.^19^

Prior studies have demonstrated inconsistent findings with respect to whether or not second doses of antibiotics are more likely to be delayed when the patient is still in the ED. Our cohort had low rates of ED boarding with only 15% of patients remaining in the ED at the time second doses were due, and this was associated with lower risk of second dose delays. This mirrors a smaller analysis conducted by Randolph et al, which included only seven patients in the ED at the time of second dose, none of whom experienced a significant delay.^20^ In contrast, 22% of patients were boarding in the ED at the time of second dose in both the Leisman and Lykins analyses and 44% were boarding in the Kemmler analysis; all three studies found significant associations between ED boarding and delays.^10,11,17^ This suggests there are important differences in care processes across various EDs that may facilitate or hinder timely administration of second antibiotic doses.

Although there are numerous studies demonstrating associations between delayed first doses of antibiotics and mortality in patients with sepsis, the impact of delays of second doses is less clear.^4–9^ There are plausible biologic reasons why second dose delays might worsen outcomes, including slowing of antibiotics’ cidal mechanisms and promoting regrowth of increasingly resistant pathogens.^21–23^ However, delays in second doses may simply be surrogates for other factors such as less attentive care overall, as speculated by Leisman et al who found a strong association between delays and worse outcomes.^10^ Kemmler et al similarly found an association between delays and mortality.^17^ In contrast, a difference in mortality was not seen by Lykins et al and in the overall cohort of Parks-Taylor et al.^11,12^ The different findings in these studies and ours might reflect inherent differences in patient populations and treatment practices and/or study methodology, including the breadth of risk adjustment.

Risk adjustment is particularly relevant to this analysis because delays in second doses of antibiotics are unlikely to be random. Indeed, significantly more patients in the non-delayed group were admitted to the ICU at the time second doses were due, while more patients in the delayed group were in non-ICUs. In addition, patients in the non-delayed group required more vasopressors, had higher baseline serum creatinine levels, and trended toward higher initial lactate levels, SOFA scores, age, and rates of bacteremia. These findings suggest that the patients in the non-delayed group were more severely ill overall, which may have contributed to more attentive care. Lykins et al similarly emphasized that more patients in their cohort were admitted to ICUs compared to the Leisman analysis and may have received more attentive care, which may account for the absence of associations between delays and outcomes in their study.^10,11^ Randolph et al also did not find differences in outcomes between patients who experienced delays and those who did not, and, like our analysis and the Lykins analysis, had high rates of ICU admission (∼68%).^11,20^

Interestingly, our subgroup analysis showed that delays in second doses of antibiotics were associated with significantly increased rates of mortality in patients admitted to non-ICUs but not ICUs. Although not directly comparable, these findings contrast with the analysis by Parks-Taylor et al that found associations between second dose delays and mortality in patients with septic shock but not sepsis without shock.^12^ It is possible that our finding simply reflects unmeasured confounding such that patients on the non-ICU wards who have delays in second doses may be sicker and require other interventions that impede timely antibiotic administrations, without the greater nursing attentiveness that is easier to provide in ICU settings. Previous studies, however, have demonstrated high mortality rates in patients with sepsis outside of ICUs, highlighting the vulnerability of this population and perhaps greater susceptibility to inadequate care compared to patients in the ICU.^24–26^ Increased mortality in hospitalized non-ICU patients has also been associated with inadequate nursing staff, which may contribute to differences in both the timeliness of second antibiotic doses and patient outcomes compared with ICUs that are more likely to have one-to-one care.^27^

Strengths of our study include rigorous antibiotic dosing assessments adjudicated by two pharmacists, limiting misclassifications between delayed and non-delayed groups, consistent sepsis identification criteria that include prospective flags for suspected sepsis (providing confidence in the timing of sepsis onset), and inclusion of a broad array of covariates for confounding adjustment. Our study also has several limitations. This was a single-center, retrospective, observational analysis. Rapid changes in renal function and clinical status, which may have impacted recommended dosing intervals, may not have been adequately captured due to the study design. In addition, baseline serum creatinine prior to admission was not collected, making it difficult to identify patients who had an acute kidney injury on admission, which may have also affected dosing intervals and delays. Our sample size was relatively modest, and so our study may have been underpowered to detect small differences in outcomes. Although we included a wide range of covariates in our models, there may be additional unmeasured confounders that influence both delays in second antibiotic doses and the risk of mortality, particularly around patients’ severity-of-illness on arrival to the hospital as well as at the time the second antibiotic doses are due.

In conclusion, over a quarter of our patients treated for sepsis in the ED experienced delays in second doses of antibiotics. Delays were more likely when the patient had transferred out of the ED before second doses were due, suggesting that transitions of care may have contributed. Although delays in second doses of antibiotics were not associated with worse outcomes in the overall cohort, hospital mortality was higher among those in the non-ICU subgroup who had delayed second doses. Further large, rigorous studies (and ideally studies that prospectively test the impact of interventions to reduce delays in second doses) are warranted to better elucidate the impact of delayed second doses of antibiotics on outcomes overall and in this patient population specifically.

## Supporting information

Cook et al. supplementary material Cook et al. supplementary material

## Supplementary material

For supplementary material accompanying this paper visit http://doi.org/10.1017/ash.2023.480. click here to view supplementary material

## Previous presentation

Preliminary findings were presented at the 2023 Society of Critical Care Medicine Critical Care Congress.
