# Supplementary material for: Prevalence, risk factors, and outcomes associated with delayed second doses of antibiotics in sepsis at a large academic medical center
Source: Antimicrob Steward Healthc Epidemiol. 2023 Nov 10;3(1):e207. doi: 10.1017/ash.2023.480 (PMC10654957; doi:10.1017/ash.2023.480)
Supplement: Cook et al. supplementary material [file S2732494X23004801sup001.docx]

**Supplementary Material**

**Prevalence, Risk Factors, and Outcomes Associated with Delayed Second Doses of Antibiotics in Sepsis at a Large Academic Medical Center**

Authors: Meghan E. Cook, PharmD^1^; Brian R. Schuler, PharmD, BCCCP^1^; Michael J. Schontz, PharmD, BCPS, BCCCP^1^; Kevin C. McLaughlin, PharmD, BCPS, BCCCP^1^; Kenneth E. Lupi, PharmD, BCPS, BCCCP^1^; Jeremy R. DeGrado, PharmD, BCCCP^1^; Chanu Rhee, MD, MPH^2,3^

^1^ Department of Pharmacy, Brigham and Women’s Hospital, Boston, MA

^2^ Department of Population Medicine, Harvard Medical School / Harvard Pilgrim Health Care Institute, Boston, MA

^3^ Division of Infectious Diseases, Department of Medicine, Brigham and Women’s Hospital, Boston, MA

**Table of Contents**

**Supplementary Table 1: Best Practice Alert (BPA) Criteria**……………………………….....3

**Supplementary Table 2. Antipseudomonal Beta-lactam Dosing**……………………………...4

**Supplementary Table 3. Vancomycin Dosing**………………………………………………….6

**Supplementary Table 4. Fluid Administration**...........................................................................7

**Supplementary Table 5. Subgroup Analysis: Patients Admitted to Non-Intensive Care Units**................................................................................................................................................8

**Supplementary Table 6. Regression Analysis Evaluating the Association Between Delayed Second Doses of Antibiotics and In-hospital Mortality in Patients Admitted to Non-Intensive Care Units**......................................................................................................................9

**Supplementary Figure 1. Vancomycin Dosing in Renal Insufficiency**...................................10

**Supplementary Table 1. Best Practice Alert (BPA) Criteria**

| **BPA** | **Criteria** |
| --- | --- |
| Sepsis | Possible*^a^* or high suspicion*^b^* of infection and ≥ 1 of the following:   - Systolic blood pressure < 90 mm Hg - Lactate > 2.0 mmol/L - Creatinine > 2.0 mg/dL (acute rise) - Initiation of mechanical or non-invasive positive pressure ventilation |
| Septic shock | Possible*^a^* or high suspicion*^b^* of infection and ≥ 1 of the following:   - Systolic blood pressure < 90 mm Hg that persists for > 3 hours - Lactate ≥ 4 mmol/L |

*^a^*Possible infection: Temperature > 100.4°F, white blood cell (WBC) count > 15 x 10^9^/L, urinalysis with > 20 WBC, culture ordered, emergency department (ED) screen positive for “possible infection”

*^b^*High suspicion of infection: Intravenous antibiotics, bands ≥ 5%, ED screen positive for “looks sick”

**Supplementary Table 2. Antipseudomonal Beta-Lactam Dosing**

| **Antibiotic** | **Creatinine clearance (mL/min)** | **Recommended dosing interval** |
| --- | --- | --- |
| Aztreonam | >50 | Q8H |
|  | 10-50 |  |
|  | <10 |  |
|  | Hemodialysis |  |
| Cefepime | ≥60 | Q8H |
|  | 30-59 | Q12H |
|  | 10-29 | Q24H |
|  | <10 |  |
|  | Hemodialysis | 1 g Q24H or 2 g post-HD |
| Ceftazidime | >50 | Q8H |
|  | 31-50 | Q12H |
|  | 16-30 | Q24H |
|  | 5-15 |  |
|  | <5 | Q48H |
|  | Hemodialysis | 1 g Q24H or 2 g post-HD |
| Imipenem-cilastatin | ≥60 | Q6H |
|  | 30-59 | Q8H |
|  | 15-29 | Q12H |
|  | Hemodialysis |  |
| Meropenem | >50 | Q8H |
|  | 26-50 | Q12H |
|  | 10-25 |  |
|  | <10 | Q24H |
|  | Hemodialysis |  |
| Piperacillin-tazobactam | >40 | Q6H |
|  | 20-40 |  |
|  | <20 |  |
|  | Hemodialysis | Q8H |

**Supplementary Table 3. Vancomycin Dosing**

| **Age (years)** | 18-49 | | | 50-79 | | ≥80 | |
| --- | --- | --- | --- | --- | --- | --- | --- |
| **Creatinine clearance (mL/min)** | ≥90 | 50-89 | ≤49 | ≥50 | ≤49 | ≥60 | ≤59 |
| **Recommended interval** | Q8H | Q12H | Q24H | Q12H | Q24H | Q12H | Q24H |

**Supplementary Table 4. Fluid Administration**

| **Variable** | **Delayed group**  **(n=123)** | **Non-delayed group (n=326)** | ***p*** |
| --- | --- | --- | --- |
| Received 30 mL/kg of fluid within 3 hours of BPA*^a^* | 39 (31.7) | 115 (35.3) | 0.48 |
| Type of crystalloid administered*^a^* |  |  |  |
| Normal saline | 100 (81.3) | 242 (74.2) | 0.12 |
| Lactated Ringer’s | 56 (45.5) | 161 (49.4) | 0.47 |
| Dextrose 5% in water | 2 (1.6) | 4 (1.2) | 0.74 |
| Other*^b^* | 11 (8.9) | 16 (4.9) | 0.11 |

BPA, best practice alert

*^a^*Data presented as n (%)

*^b^*Other fluids include dextrose 10% in water, ½ normal saline, combination fluids with dextrose and sodium chloride, sodium bicarbonate, and electrolyte containing fluids (e.g., normal saline with potassium chloride)

**Supplementary Table 5. Subgroup Analysis: Patients Admitted to Non-Intensive Care Units**

| **Outcome** | **Delayed group**  **(n=49)** | **Non-delayed group (n=122)** | ***p*** |
| --- | --- | --- | --- |
| In-hospital mortality*^a^* | 9 (18.4) | 7 (5.7) | 0.01 |
| Discharge to hospice*^a^* | 4 (8.2) | 7 (5.7) | 0.56 |
| Hospital length of stay (days)*^b^* | 5.0 [4.0-9.0] | 7.0 [4.0-11.0] | 0.08 |

*^a^*Data presented as n (%)

*^b^*Data presented as median [interquartile range]

**Supplementary Table 6. Regression Analysis Evaluating the Association Between Delayed Second Doses of Antibiotics and In-hospital Mortality in Patients Admitted to Non-Intensive Care Units**

| **Variable** | **OR (95% CI)** | ***p*** |
| --- | --- | --- |
| Delay in second dose | 4.10 (1.32-12.79) | 0.02 |
| Weight (per 1 point BMI increase) | 0.98 (0.95-1.02) | 0.31 |
| Malignancy | 1.92 (0.56-6.55) | 0.30 |
| Respiratory infection | 2.04 (0.63-6.62) | 0.23 |
| Non-ED location at time second dose due | 1.77 (0.16-20.01) | 0.65 |
| Time in ED (per hour) | 0.97 (0.82-1.15) | 0.72 |
| SOFA score (per 1 point increase) | 1.34 (1.08-1.66) | 0.01 |
| Initiation of stress dose steroids within 24 h of inclusion BPA | 1.66 (0.30-9.06) | 0.56 |

BMI, body mass index; ED, emergency department; SOFA, sequential organ failure assessment; BPA, best practice alert

**Supplementary Figure 1. Vancomycin Dosing in Renal Insufficiency**

**
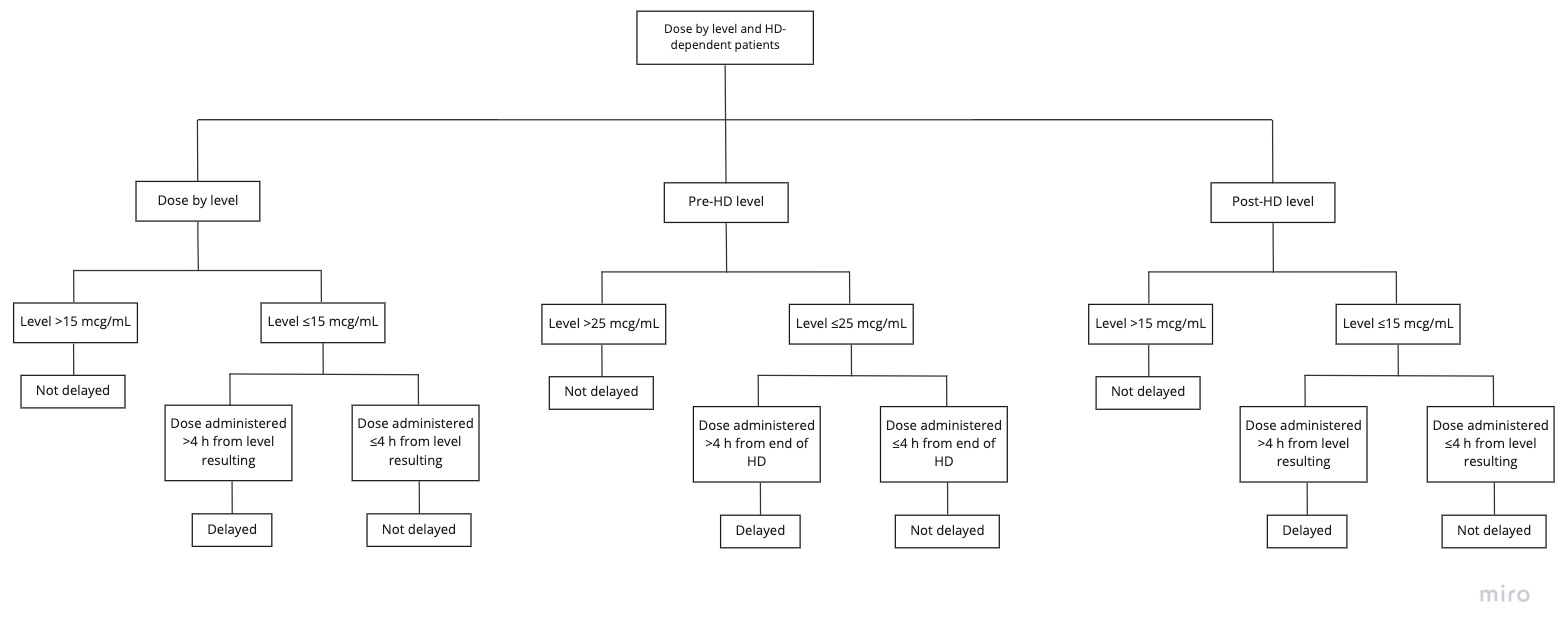
**Abbreviations: HD, hemodialysis
